# Diversification of Genes Encoding Granule-Bound Starch Synthase in Monocots and Dicots Is Marked by Multiple Genome-Wide Duplication Events

**DOI:** 10.1371/journal.pone.0030088

**Published:** 2012-01-23

**Authors:** Jun Cheng, Muhammad Awais Khan, Wen-Ming Qiu, Jing Li, Hui Zhou, Qiong Zhang, Wenwu Guo, Tingting Zhu, Junhua Peng, Fengjie Sun, Shaohua Li, Schuyler S. Korban, Yuepeng Han

**Affiliations:** 1 Key Laboratory of Plant Germplasm Enhancement and Specialty Agriculture, Wuhan Botanical Garden of the Chinese Academy of Sciences, Wuhan, Hubei, China; 2 Department of Natural Resources and Environmental Sciences, University of Illinois, Urbana, Illinois, United States of America; 3 Key Laboratory of Horticultural Plant Biology (Ministry of Education), Huazhong Agricultural University, Wuhan, Hubei, China; 4 School of Science and Technology, Georgia Gwinnett College, Lawrenceville, Georgia, United States of America; Rutgers University, United States of America

## Abstract

Starch is one of the major components of cereals, tubers, and fruits. Genes encoding granule-bound starch synthase (GBSS), which is responsible for amylose synthesis, have been extensively studied in cereals but little is known about them in fruits. Due to their low copy gene number, *GBSS* genes have been used to study plant phylogenetic and evolutionary relationships. In this study, *GBSS* genes have been isolated and characterized in three fruit trees, including apple, peach, and orange. Moreover, a comprehensive evolutionary study of *GBSS* genes has also been conducted between both monocots and eudicots. Results have revealed that genomic structures of *GBSS* genes in plants are conserved, suggesting they all have evolved from a common ancestor. In addition, the *GBSS* gene in an ancestral angiosperm must have undergone genome duplication ∼251 million years ago (MYA) to generate two families, *GBSSI* and *GBSSII*. Both *GBSSI* and *GBSSII* are found in monocots; however, *GBSSI* is absent in eudicots. The ancestral *GBSSII* must have undergone further divergence when monocots and eudicots split ∼165 MYA. This is consistent with expression profiles of *GBSS* genes, wherein these profiles are more similar to those of *GBSSII* in eudicots than to those of *GBSSI* genes in monocots. In dicots, *GBSSII* must have undergone further divergence when rosids and asterids split from each other ∼126 MYA. Taken together, these findings suggest that it is *GBSSII* rather than *GBSSI* of monocots that have orthologous relationships with *GBSS* genes of eudicots. Moreover, diversification of *GBSS* genes is mainly associated with genome-wide duplication events throughout the evolutionary course of history of monocots and eudicots.

## Introduction

Plant starch consists of a mixture of two different components, amylose (20–30%) and amylopectin (70–80%). Amylose is a linear polymer of glucose (Glc) residues joined together by α-1,4-glucosidic bonds, while amylopectin is a highly branched glucose polymer with α-1,6-glucosidic bonds linking linear chains. Amylose synthesis is relatively simple, and it is mainly catalyzed by granule-bound starch synthase (GBSS), which is encoded by the *waxy* or the *GBSS* gene. In contrast, the synthesis of amylopectin is rather complex and involves coordinated activities of different classes of enzymes, including soluble starch synthases (SSs), starch branching enzymes (SBEs), and starch debranching enzymes (DBEs) [1], [2]. Of these enzymes, SBEs introduce α-1,6-glucosidic linkages into polyglucans, while DBEs hydrolyze α-1,6-glucosidic linkages and play an important role in determining starch structure and granule characteristics during starch biolsynthesis [2]. SSs catalyze the transfer of Glc from ADP-Glcose (ADP-Glu) to non-reducing ends of glucan chains via an α-1,4-glucosidic linkage.

Genes encoding GBSS have been well characterized in starch crops as amylose content has a significant impact on physicochemical properties of starch [3]. GBSS differs from other SS isoforms due to its localization in granules and its unique functional role in starch synthesis. Not only it can transfer glucosyl residues from ADP-Glu to glucan substrates to produce relatively long-chain amylose molecules, but it also acts on side chains of amylopectin to form long chains of amylopectin [2]. The latter activity may have been the original function of GBSS, early in its evolutionary path. Moreover, amylose synthesis may be responsible for starch density, and ultimately improving the efficiency of carbon storage, thus justifying the conservation of GBSS in higher plants [2].

In cereals, GBSS consists of two isoforms, GBSSI (also known as waxy protein) and GBSSII. The *GBSSI* gene is exclusively expressed in storage tissues such as endosperms and embryos of seeds, while the *GBSSII* gene is expressed in non-storage tissues such as leaf, stem, root, and pericarp [4], [5]. Interestingly, the ratio of amylose to amylopectin in pericarps is different from that in endosperms as endosperm starch consists of both large and small granules, while pericarp starch granules are small and relatively uniform in size [6]. Thus, it is unclear if *GBSSI* and *GBSSII* genes have diverged in their functions, thereby playing different roles in amylose synthesis. Besides cereals, different isoforms of GBSS have also been reported in eudicots. For example, a pea GBSSI consists of two isoforms, GBSSIa and GBSSIb [7]. The *GBSSIa* gene is expressed predominantly in embryos with transcripts detected in leaves as well [8]. The *GBSSIb* gene is highly expressed in leaves, but with transcripts also detectable in embryos. Therefore, the expression profiles of both *GBSSIa* and *GBSSIb* genes are different from those of *GBSSI* genes in cereals.

*GBSS* genes have been widely used in many plant species to study phylogenetic and evolutionary relationships as they are of either single- or low-copy nuclear genes. For example, Evans et al. [9] used *GBSS* genes to investigate the early origins of the subfamily Maloideae, belonging to the Rosaceae family, and contrary to an earlier proposed hypothesis of wide hybridization between ancestors of two other subfamilies, they proposed its aneuploidy (x = 17) origin. They also suggested that duplication of the *GBSSI* gene must have occurred prior to diversification of Rosaceae. Moreover, *GBSS* genes have also been used to study the molecular taxonomy of Rosaceae revealing that the Pyreae tribe has originated either by autopolyploidization or by hybridization between two sister taxa, followed by diploidization, and then subsequent aneuploidization [10].

Starch is not only the main component of cereal grains, legumes, and tubers, but it is also an important component of many fruits, such as apple and banana. For example, in apple, starch begins to rapidly accumulate in young fruitlets as they reach 20 mm in diameter, and starch content can be as high as 15% before ripening [11]. Similarly, banana fruit contain about 25% starch, and may serve as raw material for producing starch [12]. Moreover, fruit starch content has a strong influence on both fruit texture and processing quality [13], [14]. For example, apples with low starch content taste sweet, and some varieties, such as Granny Smith, that contain high starch content are deemed good cooking apples. However, high starch content is a problem for processing of apple juice as it contributes to cloudiness of the apple juice. In order to degrade and eliminate insoluble starch, amylases that catalyze the hydrolysis of α-1,4-glucosidic linkages are widely used during processing of apple juice production. Although starch is one of the main components of fruits, and has a high influence on fruit quality, little is known about those genes involved in fruit starch synthesis. To date, only genes encoding SBEs have been isolated and characterized in apple [15], [16].

Recently, whole genome sequences have been released for several fruit trees such as apple [10], peach (http://www.rosaceae.org/peach/genome), and citrus (http://www.citrusgenomedb.org/). These genome sequence databases, together with integrated physical and genetic maps of the apple [17], provide unique opportunities for genome-wide investigation of genes involved in fruit starch synthesis. Identifying and characterizing starch synthesis genes, such as *GBSS*, would also afford an opportunity to pursue fundamental evolutionary studies underlying differences between monocots and eudicots. As endosperms are usually absent in mature seeds of eudicots and starch accumulates in cotyledons, it is unclear as to whether or not endosperm-specific *GBSSI* genes in monocots have diverged from *GBSS* genes in eudicots. In this study, identification and characterization of *GBSS* genes in apple, peach, and orange have been conducted, and these have been used to perform a comprehensive study to assess the evolutionary pathway of *GBSS* genes in monocots and eudicots.

## Materials and Methods

### Plant material

Young leaves, flowers, and fruits from three fruit crops, including apple (*Malus*×*domestica* Borkh.), peach [*Prunus persica* (L.) Batsch.], and orange (*Citrus sinensis* L. Osbeck) were collected and stored at −80°C until needed. Apple and peach leaves were collected in spring, while orange leaves were collected in summer. Flower buds were collected at the balloon stage. Fruits were collected at early, middle, and mature stages of development. Briefly, the early stages of peach, apple, and orange fruits were 30, 42, and 40 days after pollination (DAP), respectively. The middle stages of peach, apple, and orange fruits were 60, 105, and 150 DAP, respectively. The late stages of peach, apple, and orange fruits were 90, 168, and 220 DAP, respectively. Whole fruits were used for gene expression analysis. Both apple, belonging to the subfamily Maloideae, and peach, belonging to subfamily Amgydaloideae, are members of the Rosaceae family; while orange, belongs to the family Rutaceae. Both Rosaceae and Rutaceae families belong to rosids, and hence they are eudicots.

### Isolation of genes encoding GBSS in apple

The coding DNA sequence of an *Arabidopsis GBSS* gene (GenBank accession no. NM_103023) was BLASTed against an apple expression sequence tag (EST) database (http://titan.biotec.uiuc.edu/apple/), and three homologous EST sequences (GenBank accession nos. CN489540, CO903202, and CN496889) were identified. Primer pairs were then designed based on the three EST sequences, and used to screen an apple BAC library (cv. GoldRush) according to a PCR-based screening protocol [15]. The three primer pairs were designated as AW×1F/AW×1R, AW×2F/AW×2R, and AW×3F/AW×3R, and their sequences were as follows: 5′-GGCCTTGGTGATGTTCTTGG-3′/5′-CCTGGCACAGCAAGCTGAAG-3′, 5′-GTGGACTTGGTGATGTTCTTGG-3′/5′-CTAGAGCTGCCTGGCATAGAAG-3′, and 5′-CTGCTGTTGAGCCTGGAAGTTG-3′/5′-CGTTGCCACAGGTGGAAGTTAG-3′, respectively. The PCR program consisted of 34 cycles of 30 s at 94°C, 30 s at 60°C, 60 s at 72°C, and a final extension for 5 min at 72°C.

The primer pair AW×2F/AW×2R amplified two different size bands, and two positive BAC clones containing the two different bands, respectively, were selected for subcloning. Each of the other two primer pairs amplified only a single band, and a single positive BAC clone derived from each primer pair was subjected to subcloning. BAC DNA was extracted from a 300 ml culture using the Plasmid Midi kit (QIAGEN, Valencia, CA, USA), and BAC DNA subcloning was carried out according to Han et al. [15]. Subsequently, positive subclones were sequenced using a primer-walking strategy, and genomic DNA sequences were recovered.

### Identification of genes encoding GBSS in peach and orange

Coding sequences of genes encoding GBSS in peach and orange were downloaded from the Genome Database for Rosaceae (http://www.rosaceae.org/peach/genome) and the Citrus Genome Database (http://www.citrusgenomedb.org/), respectively. The coding sequences were then BLASTed against whole genome sequence databases to recover their corresponding genomic DNA sequences. Exon lengths were calculated by alignment of genomic DNA sequences with cDNA sequences, and introns were determined according to the “GC-AG” rule [18].

### Expression profiles of *GBSS* genes in fruit trees using real-time PCR

Total RNA was extracted according to the protocol described by Gasic et al. [19]. Approximately 3 µg of total RNA per sample was treated with DNase I (Invitrogen Life Science), and then used for cDNA synthesis. A SYBR Green-based real-time PCR assay was carried out in a total volume of 25 µL reaction mixture containing 12.5 µL of 23 SYBR Green I Master Mix (Applied Biosystems), 0.2 µM of each primer, and 100 ng template cDNA. An actin gene was used as a constitutive control.

Amplifications were performed using a 7300 Real-Time PCR System (Applied Biosystems). The amplification program consisted of one cycle of 95°C for 10 min, followed by 40 cycles of 95°C for 15 s and 60°C for 1 min. Fluorescent products were detected in the last step of each cycle. Melting curve analysis was performed at the end of 40 cycles to ensure proper amplification of target fragments. Fluorescence readings were consecutively collected during the melting process from 60°C to 90°C at a heating rate of 0.5°C s^−1^. Reaction mixtures without cDNA templates were also run as negative controls to evaluate the specificity of the real-time PCR. All analyses were repeated three times using biological replicates. Differences in cycle thresholds between target and actin genes corresponded to levels of gene expression. All primer sequences used for real-time PCR have been listed in Table 1.

**Table 1:** Primers for qPCR analysis of *GBSS* genes in apple, peach, and orange.

Species	Gene name	Forward primer	Reverse primer
Apple	*MdGBSSII-1*	5′-CTGCGGTCTCATTCAGTTGC-3′	5′-GCAACAGCTCTCTTGACAGTAGT-3′
	*MdGBSSII-2*	5′-GCCAATGGAGAAGCAGCTTGA-3′	5′-CGCCGGTTGACGCAACAATG-3′
	*MdGBSSII-3*	5′-GTTGAGCCTGGAAGTTGGTG-3′	5′-CTCTCTTGCAGCTGGAGGTTAC-3′
	*MdActin*	5′-CTACAAAGTCATCGTCCAGACAT-3′	5′-TGGGATGACATGGAGAAGATT-3′
Peach	*PpGBSSII-1*	5′-TATGGAACGGTGCCTATTGT-3′	5′-CATCTGCTGGGTCAACTTCA-3′
	*PpGBSSII-2*	5′-ATGCGTTACGGAACTGTGC-3′	5′-TACATCCGCAGGATCAACAA-3′
	*PpActin*	5′-GGTGTGACGATGAAGAGTGATG-3′	5′-TGAAGGAGAGGGAAGGTGAAAG-3′
Citrus	*CSGBSSII-1*	5′- GCAGATTTCCCTTCGCAGACT -3′	5′- AAAGCCCCCCTGAAATCATC -3′
	*CsGBSSII-2*	5′-GGGACTTGGTGATGTTCTTGGA-3′	5′-TCATGACACGGTGCCCATT-3′
	*CsActin*	5′-CCAAGCAGCATGAAGATGAA-3′	5′-ATCTGCTGGAAGGTGCTGAG-3′

### Estimation of the divergence time of *GBSS* genes

The coding DNA sequences were aligned using MUSCLE (multiple sequence comparison by log-expectation) [20] and integrated in MEGA5. This was used for dating of divergence events and relationships among GBSS genes. The molecular clock was calibrated using two calibration points; divergence of rice-maize (31.0±6.0 MYA) as well as that of apple-orange (106.0±4.0 MYA). These calibrations served as landmarks to assess the posterior distribution of estimated divergence time points among all samples used. A Bayesian Markov chain Monte Carlo (MCMC) analysis implemented in Beast 1.5.4 [21] was used to estimate divergence dates. A relaxed molecular clock approach with an uncorrected log-normal distribution model of rate of variation, Yule model of speciation for branching rates, and the Hasegawa, Kishino, and Yao (HKY) model of nucleotide substitution [22] with four rate categories was used. This analysis included four independent runs, each with 20 million MCMC steps, and sampled every 1000 generations. Results from all runs were combined and summarized with LOGCOMBINER ver. 1.4.6 [21], and used to check for convergence and for estimating efficient sample size (ESS), mean, and 95% credible intervals of each parameter, using Tracer v1.5 [23]. A maximum clade credibility tree was generated using Tree Annotator program, and the tree was graphically visualized and drawn using FigTree v1.3.1 software [21].

## Results

### Genomic sequences of *GBSS* genes in apple, peach, and orange

**Figure 1:**
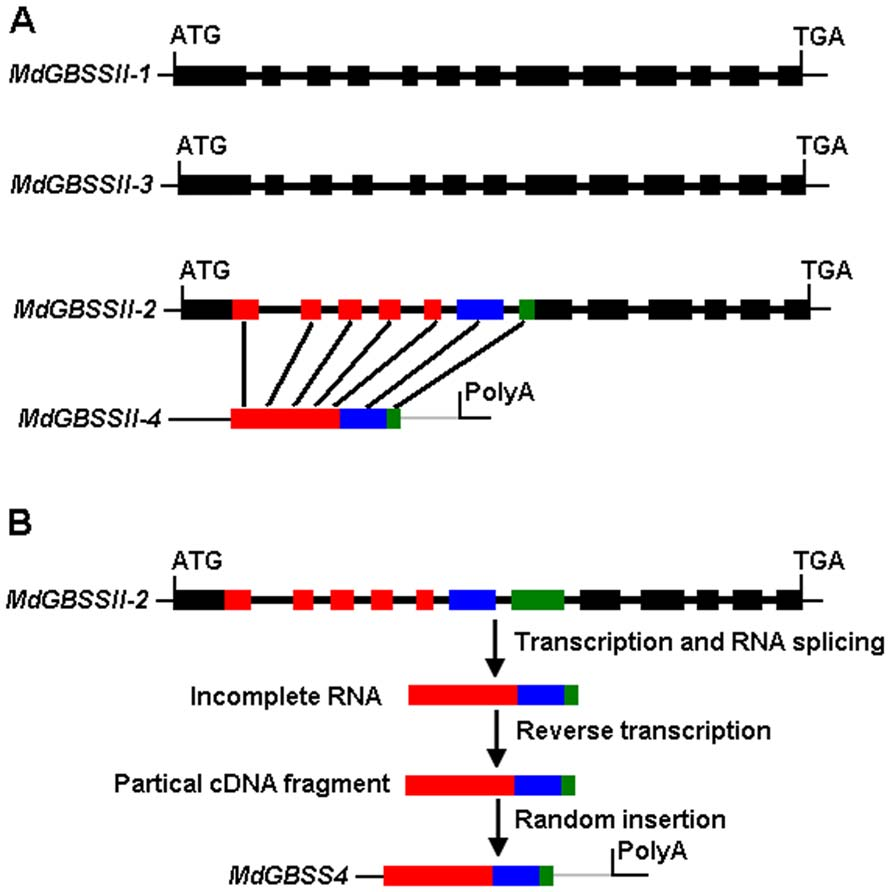
Structural relationships among genes encoding GBSS in apple. A, Structural organizations of *MdGBSSII-1* to *MdGBSSII-4*. B, Possible mechanism underlying the generation of retropseudogene of *MdGBSSII-4*. Solid boxes indicate exons, and bold lines represent introns. Exons of the *MdGBSSII-3* gene corresponding to sequences of *MdGBSSII-4* gene are depicted in the same color. The gray line indicates repetitive sequences in the apple genome.

In apple, three genes encoding GBSS, designated as *MdGBSSII-1, MdGBSSII-2*, and *MdGBSSII-3* with GenBank accession nos. JN187080, JN187081, and JN187082, respectively, were isolated and sequenced in this study. *MdGBSSII-1* and *MdGBSSII-3* are very closely related and share 92% identity in coding DNA sequences. Both *MdGBSSII-1* and *MdGBSSII-3* show 74% identity in coding DNA sequences with *MdGBSSII-2*. These three genes are conserved in their genomic structures (Table 2). Both *MdGBSSII-1* and *MdGBSSII-3* contain 13 exons (Fig. 1A), with all exons, except for exon 9, having identical lengths. *MdGBSSII-2* consists of 12 exons, with exons 2 to 5, 8, and 10 to 13 being of identical lengths to those of either *MdGBSSII-1* or *MdGBSSII-3*. However, exon 6 of *MdGBSSII-2* is equal to the combined lengths of exons 6 and 7 of *GBSS* genes of both eudicots and monocots, thus indicating that an intron loss event must have occurred during the evolution of the apple *MdGBSSII-2* gene. Moreover, a pseudogene, designated as *MdGBSSII-4*, has also been isolated in this study. This *MdGBSSII-4* gene consists of a GBSS-like fragment, a repetitive sequence, and a typical poly (A) tail at its 3′ end (Fig. 1A). The GBSS-like fragment of 804 bp in size shows a high nucleotide sequence identity (96%) with seven combined exons of the *MdGBSSII-2* gene, including a partial sequence of exon 1, exons 2 to 6, and a partial sequence of exon 7, while it has only 75% nucleotide sequence identity with coding sequences of either *MdGBSSII-1* or *MdGBSSII-3*. Therefore, *MdGBSSII-4* may have originated from *MdGBSSII-2* via a retroposition mechanism (Fig. 1B) [24]. In addition, DNA sequences of *MdGBSSII-1*, *MdGBSSII-2*, and *MdGBSSII-3* were BLASTed against the apple genome sequence database, and a *GBSS* pseudogene, designated *MdGBSSII-5*, was identified. The *MdGBSSII-5* contains frame shifts and stop codons in exon 6.

**Table 2:** Exon lengths (bp) of genes encoding GBSS in plants*.

No.	Apple			Peach		Citrus*		Rice		Maize		*Arabidopsis*	Potato	Wheat	Barley
	*MdGBSSII-1*	*MdGBSSII-2*	*MdGBSSII-3*	*PpGBSSII-1*	*PpGBSSII-2*	*CsGBSSII-1*	*CsGBSSII-2*	*OsGBSSI*	*OsGBSSII*	*ZmGBSSI*	*ZmGBSSII*	*AtGBSS*	*StGBSS*	*TaGBSSI*	*HvGBSSI*
1	351	357	351	351	366	345	345	339	333	321	333	342	333	321	318
2	81	81	81	81	81	81	81	81	81	81	81	81	81	81	81
3	99	99	99	99	99	99	99	99	99	99	99	99	99	99	99
4	90	90	90	90	90	**154**	90	90	90	90	90	90	90	**154**	**154**
5	64	64	64	64	64		64	64	64	64	64	64	64		
6	101	**211**	101	101	101	101	101	101	101	101	101	101	101	101	101
7	110		110	110	110	110	110	110	110	110	110	110	110	**354**	**354**
8	244	244	244	244	244	244	244	244	247	244	247	244	244		
9	180	177	174	177	177	177	177	177	177	183	177	177	177	180	180
10	192	192	192	192	192	192	192	192	192	192	192	**279**	192	192	192
11	87	87	87	87	87	87	87	87	87	87	87		87	87	87
12	129	129	129	129	129	129	129	129	129	129	129	129	129	129	129
13	117	117	117	117	117	117	117	117	117	117	120	117	117	117	117

*The first and last exons are represented as the encoding sequence, and GenBank accession nos. are the same as those used in Fig. 3. Those exons that are derived from two combined exons are highlighted in bold.

In both peach and orange genomes, there are two genes encoding GBSS. The peach *PpGBSSII-1* and *PpGBSSII-2* share 74% identity in coding DNA sequences. Each of *PpGBSSII-1* and *PpGBSSII-2* contain 13 exons, with exons 2 through 13 being of identical lengths, respectively (Table 2). Similarly, orange *CsGBSSII-1* and *CsGBSSII-2* share 73% identity in coding DNA sequences. *CsGBSSII-1* and *CsGBSSII-2* consist of 12 and 13 exons, respectively, with most of exons 1 to 3 and 6 to 13 being of identical lengths, respectively (Table 2). It is interesting to note that exon 4 of *CsGBSSII-1* is equal in length to the combined exons 4 and 5 of *CsGBSSII-2*, also suggesting that an intron loss event must have occurred during the evolutionary path of the *CsGBSSII-1* gene.

### Expression profiles of *GBSS* genes in apple, peach, and orange

**Figure 2:**
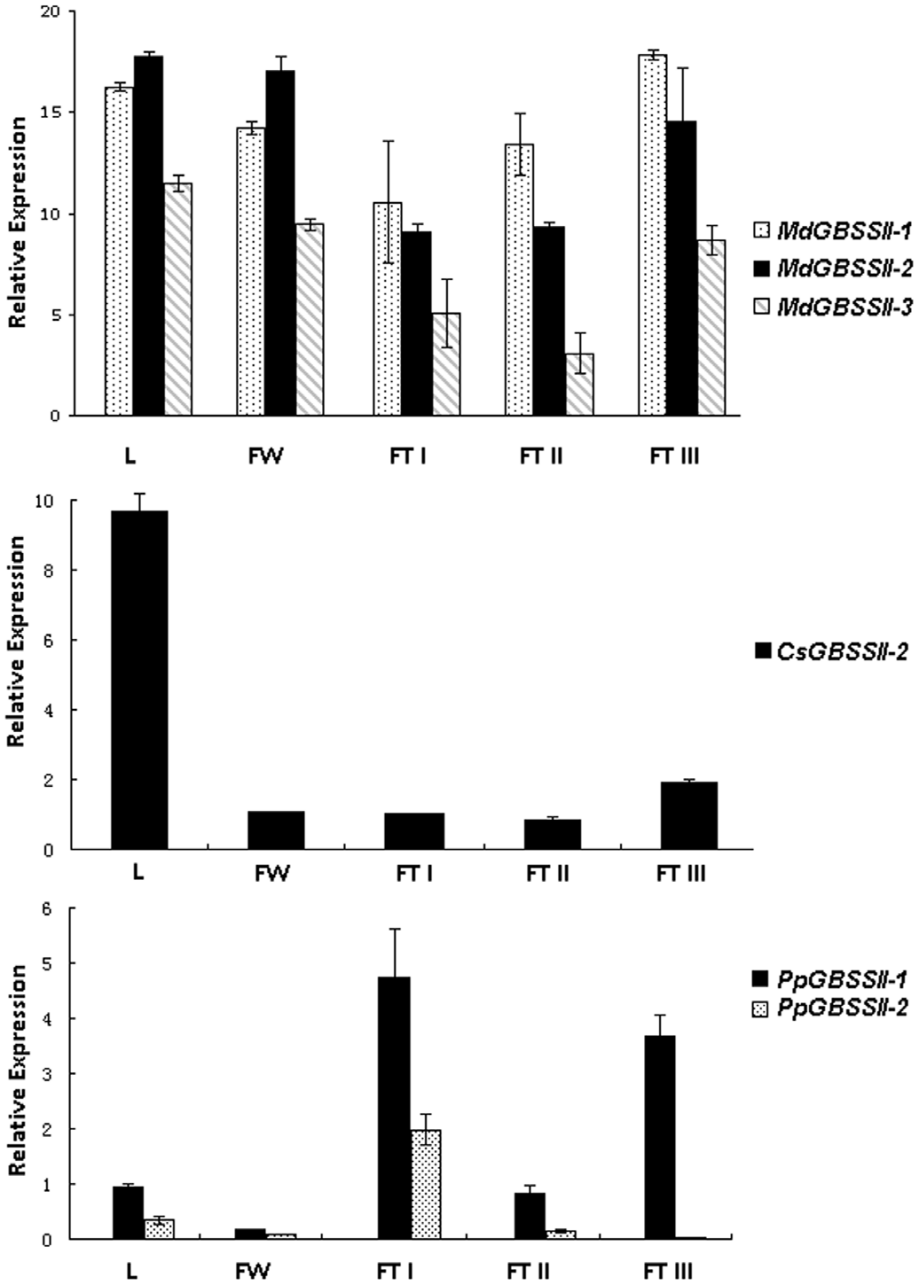
Analysis of expression profiles of genes encoding GBSS in apple, peach, and orange using real-time PCR. L, young leaves; FW, flowers; FTI, young fruitlets; FTII,fruits at mid- developmental stage; and FTIII, fruits at maturity.

 Real-time PCR results of *GBSS* genes of apple cv. Golden Delicious revealed that the three *GBSS* genes, *MdGBSSII-1* to *MdGBSSII-3*, were expressed in all analyzed tissues, including young leaves, flowers, and fruits (Fig. 2). Overall, *MdGBSSII-3* transcripts were the lowest in all analyzed tissues when compared to those of both *MdGBSSII-1* and *MdGBSSII-2*. Moreover, expression levels of *MdGBSSII-2* in both leaves and flowers were higher than those of *MdGBSSII-1* and *MdGBSSII-3*. Whereas, expression levels of *MdGBSSII-1* in apple fruit at different stages of development were higher than those of either *MdGBSSII-2* or *MdGBSSII-3*. Throughout apple fruit development, levels of both *MdGBSSII-1* and *MdGBSSII-2* transcripts were relatively low at the young fruitlet stage, but then increased as fruit continued to develop and reached maturity.

When transcripts of *GBSS* were investigated in orange cv. Honganlucheng, expression levels of *CsGBSSII-1* were too low for detection in all analyzed tissues, including leaves, flowers, and fruits. In contrast, *CsGBSSII-2* was expressed in all analyzed tissues. The expression level of *CsGBSSII-2* in leaves was higher than those in either flowers or fruits (Fig. 2).

Analysis of GBSS expression in peach cv. Huyou 018 revealed that transcripts of *PpGBSSII-1* and *PpGBSSII-2* were detected in all analyzed tissues, and these were higher in fruits than in either leaves or flowers (Fig. 2). *PpGBSSII-2* was primarily expressed in leaves and young fruitlets; however, its levels of expression in both leaves and fruits were lower than those of *PpGBSSII-1*. Transcripts of *PpGBSSII-2* were relatively high at the young fruitlet stage, but then decreased as fruit continued to develop and reached maturity. *PpGBSSII-1* transcripts were relatively low in fruit at middle development stage, during the period of endocarp hardening, as compared to early and mature stages of development.

### Genomic structure of *GBSS* genes in monocots and eudicots

Genomic sequences of genes encoding GBSSI and GBSSII have been isolated from both eudicots, such as *Arabidopsis* and potato, as well as monocots, such as rice, maize, wheat, and barley. All these *GBSS* genes, including rice *OsGBSSI* and *OsGBSSII*, maize *ZmGBSSI* and *ZmGBSSII*, potato *StGBSS*, apple *MdGBSSII-1* and *MdGBSSII-3*, peach *PpGBSSII-1* and *PpGBSSII-2*, and orange *CsGBSSII-2*, contain 13 exons, and their exon profiles are strikingly similar, with exons 2 through 7 and 10 through 13 being of identical lengths (Table 2). Whereas, wheat *TaGBSSI*, barley *HvGBSSI*, and *Arabidopsis AtGBSS* have either one or two less exons compared with *GBSSI* or *GBSSII* genes in other plants such as rice, maize, and peach. Exon 4 of *GBSSI* in both wheat and barley is equal in length to the two combined exons 4 and 5 of *GBSSI* or *GBSSII* in rice and maize. Likewise, exon 6 of *GBSSI* in wheat and barley is equal in length to the two combined exons 7 and 8 of *GBSSI* or *GBSSII* in rice and maize. Thus, two introns must have been lost during the evolutionary process of *GBSSI* genes in wheat and barley. Moreover, exon 10 of *Arabidopsis AtGBSS* is equal in length to the two combined exons 10 and 11 of *GBSSI* or *GBSSII* in other plants, suggesting that a single intron must have been lost during the evolutionary path of *AtGBSS* genes. Taken together, these results indicate that *GBSS* genes in both eudicots and monocots are conserved in genomic structure, and they have all evolved from a common ancestor.

### Timeline of *GBSS* evolution in higher plants

**Figure 3:**
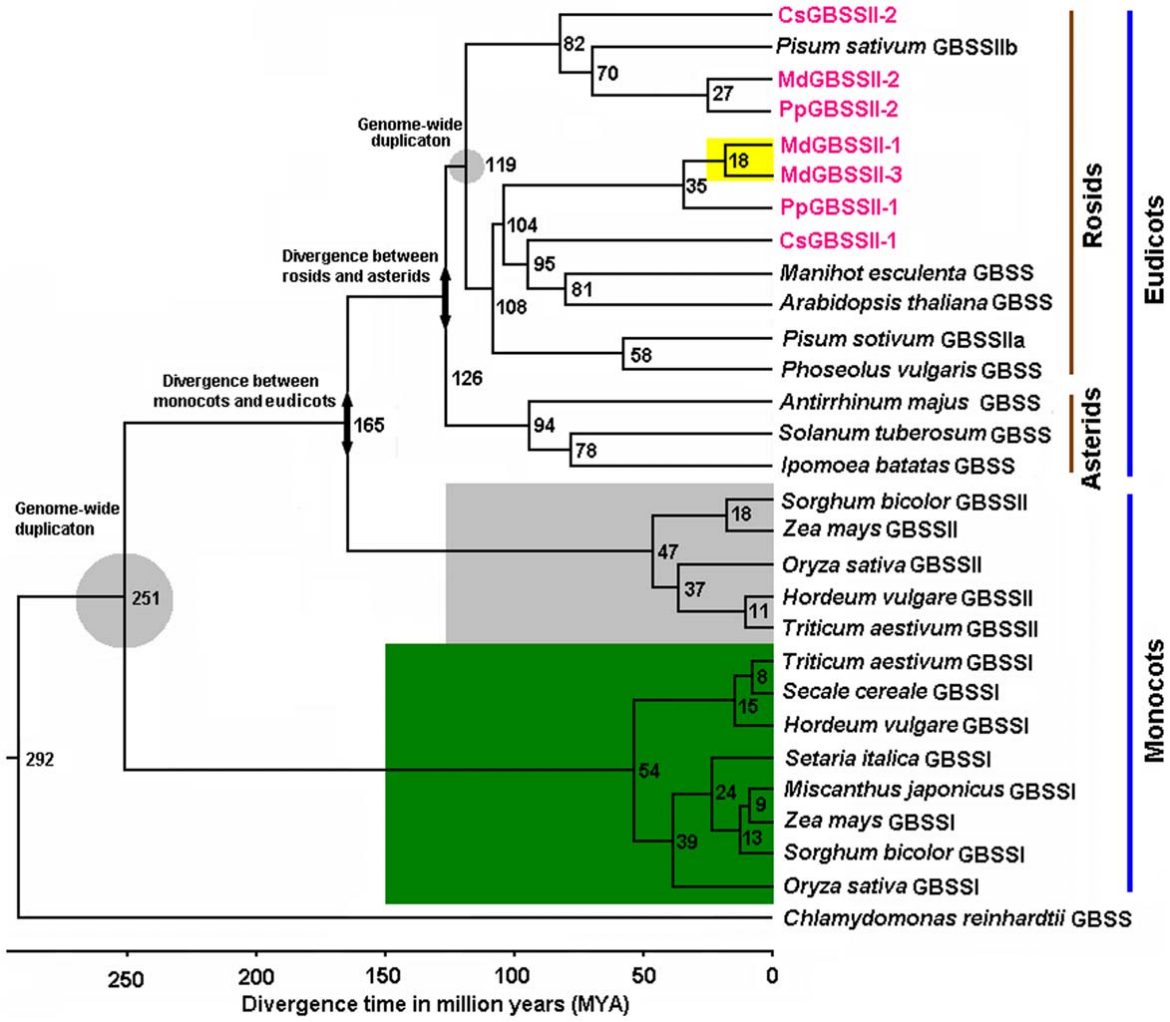
Estimated divergence time between *GBSS* genes in plants based on aligned nucleotide sequences using Bayesian MCMC analysis. A relaxed molecular clock approach, uncorrected lognormal distribution model, Yule model of speciation process, and the HKY model of nucleotide substitution were used. The Beast 1.5.4. was run for four independent times, each with 20,000,000 MCMC steps, and sampled once every 1000 generation. The molecular clock was calibrated using the divergence of rice-maize (31±6.0 MYA) and apple-orange (106±4.0 MYA). Numbers at each node represent estimated time in million years (MYA). The gray circle shows the duplication event in the ancestors of both monocots and dicots resulting in 2 copies of *GBSS* gene while divergence between monocots and dicots and rosids and asterids are represented by double-headed arrows. Clades shaded in green and grey correspond to *GBSSI* and *GBSSII* genes in monocots, respectively. Yellow shade indicates *GBSS* gene duplication in the apple genome. *GBSS* genes identified in this study are highlighted in red. The GenBank accession numbers of previously reported *GBSS* genes are listed as follows: *Manihot esculenta* GBSS (X74160), *Arabidopsis thaliana* GBSS (AY123983), *Antirrhinum majus* GBSS (AJ006293), *Ipomoea batatas* GBSS (IBU44126), *Solanum tuberosum* GBSS (EU403426), *Phaseolus vulgaris* GBSS (AB029546), *Pisum sativum* GBSSIIa (X88789), *Pisum sativum* GBSSIIb (AJ345045), *Triticum aestivum* GBSSII (AF109395), *Triticum aestivum* GBSSI (AB019624), *Hordeum vulgare* GBSSII (AK368223), *Hordeum vulgare* GBSSI (AF474373), *Zea mays* GBSSII (NM_001112569), *Zea mays* GBSSI (NM_001111531), *Sorghum bicolor* GBSS2 (EF472254), *Sorghum bicolor* GBSSI (SBU23945), *Oryza sativa* GBSSII (AY069940), *Oryza sativa* GBSSI (AF141955), *Miscanthus japonicus* GBSSI (AF446083), *Setaria italica* GBSSI (AB089141), *Secale cereale* GBSSI (FJ491377), *Citrus sinensis* CsGBSSII-1 (JN936858), *Citrus sinensis* CsGBSSII-2 (JN936859), *Prunus persica* PpGBSSII-1 (JN936860), *Prunus persica* PpGBSSII-2 (JN936862), and *Chlamydomonas reinhardtii* (AF026420).

The evolutionary history of *GBSS* genes was assessed across monocots and eudicots, revealing multiple duplication events (Fig. 3). The ancestral *GBSS* gene in early monocots and dicots must have undergone duplication, ∼251 million years ago (MYA), to generate the two gene families *GBSSI* and *GBSSII*. The next major divergence of *GBSS* must have occurred when monocots and dicots split ∼165 MYA. As monocots have not undergone any major events until speciation of grasses, beginning ∼47–54 MYA, this must have contributed to recovery of various orthologs of *GBSSI* and *GBSSII*. Subsequently in dicots, *GBSS* once again must have diverged when rosids and asterids split from one another ∼126 MYA. While *GBSS* in asterids must have retained isoforms of *GBSS* genes resulting from that divergence, a major duplication event that has occurred ∼119 MYA must have also generated two isoforms of *GBSS*. Any further divergence has represented speciation events. These results suggest that there are no new additional isoforms of *GBSS* that have evolved following the well-known pome fruit origin event that has occurred ∼27 to 35 MYA. However in apple, a duplication event that has occurred ∼18 MYA has generated paralogs of *GBSS*. Meanwhile, one of the dicot-specific *GBSS* isoforms has been lost in *Arabidopsis*.

In eudicots, multiple *GBSS* genes within the same species exhibit relatively high sequence divergence although they still have higher identities with each other than with either *GBSSI* or *GBSSII* genes from monocots. For example, apple *MdGBSSII-1* and *MdGBSSII-2* share 74% identity in amino acid sequences, while they show 59 to 68% identity in amino acid sequences with either *GBSSI* or *GBSSII* of monocots. Also, peach *PpGBSSII-1* and *PpGBSSII-2* share 76% identity in amino acid sequences, but show 60 to 66% identity in amino acid sequences with *GBSSI* or *GBSSII* of monocots. Again, orange *CsGBSSII-1* and *CsGBSSII-2* share 71% identity in amino acid sequences, while they show 59 to 66% identity in amino acid sequences with *GBSSI* or *GBSSII* of monocots. Finally, pea *PsGBSSIa* and *PsGBSSIb* share 68% identity in amino acid sequences, yet they have only 55 to 64% identity in amino acid sequences with *GBSSI* or *GBSSII* from monocots.

Overall, these findings demonstrate that genes encoding GBSS in plants are grouped into two clusters, GBSSI and GBSSII, and that genes encoding GBSSI are exclusive to monocots. Moreover, *GBSS* genes in eudicots share a common ancestor with *GBSSII* rather than with *GBSSI* of monocots.

## Discussion

### Diversification of *GBSS* genes in monocots and eudicots is mainly associated with genome duplication events

It has been well documented that a whole-genome duplication (WGD) event must have occurred in an angiosperm ancestor 150 to 270 MYA [25]. Thus, duplication of the ancestral *GBSS* gene must have likely been the result of the WGD of the angiosperm ancestor. Based on findings in this study, the *GBSS* gene must have subsequently undergone duplication into the two families of *GBSSI* and *GBSSII* ∼251 MYA (Fig. 3). In monocots, *GBSSI* and *GBSSII* have been isolated from many plants, including rice, wheat, barley, maize, sorghum, foxtail millet, and miscanthus [26], [27], [28], [29], [30], [31]. In this study, divergence of *GBSSI* and *GBSSII* in monocots must have occurred 47 to 54 MYA. This result is consistent with a previous report suggesting that radiation of grasses has occurred 50 to 70 MYA [32]. Moreover, findings in this study have revealed that all *GBSS* genes in eudicots belong to the *GBSSII* family. As *GBSSI* is absent in eudicots, this suggests the *GBSSI* subfamily has been lost during the evolutionary process of eudicots.

 It has been reported that WGD must have also occurred in an ancestral eudicot ∼125 MYA [25]. Based on this study, the *GBSS* gene in rosids must have undergone duplication into two isoforms ∼119 MYA. For example, there are two copies of *GBSS* genes in genomes of both peach and orange. Moreover, Edwards et al. [7] have confirmed presence of two isoforms of GBSS in pea. Therefore, duplication of the *GBSS* gene in rosids must have also resulted from the WGD event. Thus, duplication of the *GBSS* gene has likely occurred prior to divergence of rosids from asterids. The *Arabidopsis* has undergone WGD [33], and thus the key model eudicot plant species *A. thaliana* should also contain two copies of *GBSS* genes. Upon search, only a single *GBSS* gene has been identified in the *Arabidopsis* genome (http://www.arabidopsis.org/). In a previous study, we have reported that a starch branching enzyme encoding gene, *SBEI*, has been lost in the *Arabidopsis* genome [15]. Thus, it is quite likely that a *GBSS* gene has also been lost during the speciation of *Arabidopsis* as silencing of a *GBSS* gene is not lethal.

It is worth noting that there are more than two copies of the *GBSS* gene found in apple. DNA sequences of three genes, *MdGBSSII-1 to MdGBSSII-3*, have been BLASTed against the apple genome sequence database, and a *GBSS* pseudogene *MdGBSSII-5* has been identified on chromosome 16. The *MdGBSSII-5* shares 92% identity in genomic DNA sequence with *MdGBSSII-2*, located on chromosome 7. These BLAST results have also indicated that *MdGBSSII-1* and *MdGBSSII-3* are located on homologous chromosomes 6 and 14, respectively. As the apple genome has a polyploidy origin [34], and chromosome pairs 6–14, 1–7, and 9–17 show homoeology [17], the gene pair *MdGBSSII-1*/*MdGBSSII-3* is most likely derived from a whole-genome duplication, while the gene pair *MdGBSSII-2*/*MdGBSSII-5* must have probably resulted from whole-genome duplication followed by chromosome translocation. Therefore, evolution of the two *GBSS* gene pairs, *MdGBSSII-1*/*MdGBSSII-3* and *MdGBSSII-2*/*MdGBSSII-5*, is likely attributed to two WGD events. This finding suggests that the second WGD event must have occurred very recently, ∼18 MYA, and later than the archeobotanical dates of 48 to 50 MYA [35]. This observed difference may be attributed to the fact that our estimation is based on segmental duplication rather than WGD of the apple genome. Taken together, these findings suggest that expansion of *GBSS* genes in both monocots and eudicots is mainly due to the WGD event.

### Genes encoding GBSS in eudicots have an orthologous relationship with *GBSSII* rather than *GBSSI* genes in monocots

Comparisons of coding sequences and expression profiles of GBSS genes between species of monocots and eudicots strongly suggest that *GBSSII* rather than *GBSSI* genes in monocots have orthologous relationships with *GBSS* genes in eudicots. *GBSSI* and *GBSSII* genes in monocots have diverged greatly, sharing ∼63% identity in nucleotide sequences. *GBSSI* genes in monocots and *GBSSII* genes in eudicots share 59–67% identity in coding DNA sequences. Whereas, coding sequences of *GBSSII* genes in monocots share 67–70% identity with those of *GBSS* genes in eudicots. Thus, *GBSS* genes in eudicots show slightly higher identity in nucleotide sequences with *GBSSII* genes than with *GBSSI* genesof monocots.

In monocots, *GBSSI* genes are exclusively expressed in reproductive tissues such as endosperm and pollen; whereas, *GBSSII* genes are expressed in both vegetative and reproductive tissues including leaf, stem and pericarp [4], [5]. In this study, *GBSS* genes in apple, peach, and orange are expressed in both vegetative and reproductive tissues, including leaves, flowers, and fruits, and thus are similar to *GBSSII* genes in monocots. Moreover, while the pea *GBSSII* gene is expressed in all tissues such as leaves, pods, roots, embryos, stipules, and flowers, the pea *GBSSI* gene is expressed in leaves, pod, roots, and embryos, but not in flowers and stipules [36]. Overall, expression profiles of *GBSS* genes in eudicots are more similar to those of *GBSSII* than to *GBSSI* genes of monocots. Based on divergence time estimation, *GBSS* genes in eudicots are also more closely related to *GBSSII* genes than to *GBSSI* genes of monocots (Fig. 3).

### Expression of *GBSS* genes is likely related to starch accumulation in fruit

In this study, it is observed that expression of *GBSS* genes is likely related to starch content in fruit, and that high levels of expression of *GBSS* genes contribute to starch accumulation in apple fruit. As starch accumulates in apple fruit throughout development, it is a major component of apple fruit [11]. In contrast, sugars are the main carbohydrates in both peach and orange fruits [37], [38]. In apple fruit, all three *GBSS* genes, *MdGBSSII-1* to *MdGBSSII-3*, are highly expressed during all developmental stages. In peach fruit, the *PpGBSSII-1* gene is highly expressed during early and late stages of development; while, the *PpGBSSII-2* gene is expressed only in early developing fruit, but its transcripts are not detectable during later stages of fruit development. In orange fruit, the *CsGBSSII-2* gene is only weakly expressed throughout fruit development, while the *CsGBSSII-1* transcript is not detected at any stage of fruit development. These findings suggest that different *GBSS* genes in eudicot fruit species, belonging to Rosaceae (apple and peach) and Rutaceae (orange) families, contribute to starch accumulation in fruit, but at different stages of development and at varying levels.

Divergence analysis has revealed that the orange *CsGBSSII-2* and the pea *PsGBSSIb* genes must have shared a common ancestor ∼82 MYA (Fig. 3). Both genes are predominately expressed in leaves [7]. However, the orange *CsGBSSII-1* gene shares higher nucleotide sequence identity with the pea *PsGBSSIa* gene, which is predmoninately expressed in embryos [8], than with the pea *PsGBSSIb* gene. In this study, when expression profiles of *CsGBSSII-1* are investigated in two citrus cultivars, Honganlucheng and Clementine, a tangerine, transcripts of *CsGBSSII-1* are either non-detectable or very weak, respectively. Moreover, when the coding sequence of *CsGBSSII-1* is blasted against the citrus EST database at NCBI, no matching ESTs are identified. Given the fact that citrus fruits accumulate little amount of starch, it is reasonable to suggest that throughout the evoluationary history of citrus, the orange *CsGBSSII-1* gene has lost its function due to absence of selection pressure. It is likely that this loss of function of the *GBSS* gene has also occurred in potato as well.

### Retropseudogenes derived from mRNA encoding GBSS in apple

In this study, we have reported for the first time on the presence of retropseudogenes in the apple genome. Retropseudogenes, also known as processed pseudogenes, are generated by reverse transcription of processed mRNAs that randomly insert the resulting cDNAs into the genome [39]. These retropseudogenes contain several distinguished features, including Poly-(A) tails at their 3′ ends, lack of promoters and introns, and presence of flanking short direct repeats [40]. To date, retropseudogenes have been extensively investigated in the human genome [41], [42], [43] with only few reports on retropseudogenes in plants.

The apple *MdGBSSII-4* lacks introns and posseses a Poly-(A) tail at its 3′ end. It shares 75%, 96%, 75%, and 92% identity in nucleotide sequences with *MdGBSSII-1*, *MdGBSSII-2*, *MdGBSSII-3*, and *MdGBSSII-5*, respectively. Thus, it is apparent that *MdGBSSII-4* shows higher nucleotide sequence identity with *MdGBSSII-2* than with *MdGBSSII-I*, *MdGBSSII-3*, or with the pseudogene *MdGBSSII-5*. Therefore, *MdGBSSII-4* represents a retropseudogene found in the apple genome, and it must have been derived from mRNA of the functional gene *MdGBSSII-2* through the mechanism of retroposition (Fig. 1B) [44]. When the DNA sequence of the retropseudogene *MdGBSSII-4* is blasted against the apple genome sequence database, two additional homologues of *MdGBSSII-4* are detected in the apple genome. *MdGBSSII-4* and its two homologues share 95% identity in nucleotide sequences, and they are all located on chromosome 9. The two homologues are clustered within a 26 kb region, and they are ∼14 Mb apart from *MdGBSSII-4*. Multiple copies of the retropseudogene found on the same chromosome may be attributed to the following two factors. First, sequences of *MdGBSSII-4* have been compared with the apple EST database, identifying an EST (GenBank accession number EG631210) that is almost identical with one of the *MdGBSSII-4* homologues. The expressed *MdGBSSII-4* homologue may be located downstream of a promoter region, and its mRNA transcript can thus be transcribed and subsequently incorporated back into the apple genome, leading to expansion of retropseudogenes. Second, expansion of the *MdGBSSII-4* may be due to segmental duplication, resulting in two retropseudogenes that are clustered together [17].

### *GBSSI* and *SBEIIb* genes may represent unique evolutionary processes of the starch biosynthetic pathway in grasses

Findings in this study as well as those reported in previous studies suggest that *GBSSI* and *SBEIIb* genes, exclusively expressed in endosperms of monocots, may play critical roles in the evolution of the starch biosynthesis pathway in cereal grains. Earlier, it has been reported that there is an evolutionary disparity in *SBEII* genes between monocots and eudicots [16]. In monocots, the *SBEII* gene has duplicated into *SBEIIa* and *SBEIIb* prior to the radiation of grasses (Poaceae); while in eudicots, the duplication of *SBEII* genes has followed the process of speciation. *SBEIIa* in monocots has shown orthologous relationships with *SBEII* genes in eudicots; moreover, *SBEIIb* in monocots is likely derived from the duplication of *SBEIIa*. However, *SBEIIb* may have diverged in its function from *SBEIIa*, wherein *SBEIIb* plays an important role in branching short chains during starch biosynthesis in endosperms, while *SBEIIa* is responsible for starch-branching activity in leaves [45]. As reported in this study, *GBSSII* genes in monocots and *GBSS* genes in eudicots have orthologous relationships, suggesting these are derived from the common ancestor of monocots and eudicots. Our finding that *GBSSI* is absent in eudicots may be due to the fact endosperms are usually absent in mature eudicot seeds. Thus, GBSSI has remained specific to monocots, and leading to a separate evolutionary path for GBSS genes in eudicots. *GBSSI* and *GBSSII* genes in monocots may have also diverged in function as well. *GBSSI* transcripts are predominantly expressed in endosperm and pollen, while *GBSSII* transcripts are expressed in non-storage tissues, including leaf, stem, and pericarp [4]. As stated above, endosperms are usually absent in mature seeds of eudicots and starch accumulates in cotyledons as seeds develop. This evolutionary path can be attributed to the fact that amylose synthesis can contribute to increased density of starch, and thereby leading to increased efficiency in carbon storage [2].

Besides *GBSS* and *SBE* genes, *SS* and *DBE* genes are also involved in starch synthesis. Based on the phylogenetic analysis of *SS* and *DBE* genes conducted in this study, there are no differences in duplication patterns between monocots and eudicots (data not shown). For example, DBE in plants can be divided into two families, pullulanase (PULL) and isoamylase (ISA); and the latter has three ISA isoforms, including ISA1, ISA2, and ISA3 [46], [47]. Although the four types of *DBE* genes, including *PULL*, *ISA1*, *ISA2*, and *ISA3*, are present in both eudicots and monocots, *DBE* genes of the same type, from both monocots and eudicots, are clustered together. It is likely that SS has four isoforms in plants, including SSI, SSII, SSIII, and SSIV. *SS* genes encoding the same isoform from monocots and eudicots are also clustered together. Therefore, *GBSSI* and *SBEIIb* genes may correspond to unique evolutionary processes undertaken in the starch biosynthetic pathway in grasses (Poaceae). It may be feasible to produce novel starch by transforming *GBSSI* and/or *SBEIIb* genes of monocots into eudicot crops, such as those of cassava and potato. In addition, ISA1 and ISA2 proteins function together in a complex in tuber, leaf, and cotyledon of dicots [48], while both homomeric ISA1 and heteromeric ISA1/ISA2 complexes have been reported in endosperm of monocots [49]. This result suggests that *ISA1* gene may also have diverged in functionality between monocots and dicots.
